# Evaluating the User Experience and Usability of Game-Based Cognitive Assessments for Older People: Systematic Review

**DOI:** 10.2196/65252

**Published:** 2025-06-11

**Authors:** Rhys Mantell, Ye In Jane Hwang, Matthew Dark, Kylie Radford, Michael Kasumovic, Lauren Monds, Peter W Schofield, Tony Butler, Adrienne Withall

**Affiliations:** 1 School of Population Health Faculty of Medicine and Health University of New South Wales (UNSW) Sydney Australia; 2 Ageing Futures Institute University of New South Wales Sydney Australia; 3 Neuroscience Research Australia (NeuRA) Sydney Australia; 4 School of Psychology, Faculty of Science University of New South Wales Sydney Australia; 5 School of Biological, Earth & Environmental Sciences Faculty of Science University of New South Wales Sydney Australia; 6 Arludo Holdings Pty Ltd Sydney Australia; 7 Specialty of Addiction Medicine Central Clinical School The University of Sydney Sydney Australia; 8 Neuropsychiatry Service, Hunter New England Health Newcastle Australia; 9 School of Medicine & Public Health University of Newcastle Newcastle Australia

**Keywords:** serious games, gamification, cognitive assessment, usability, user experience, aging

## Abstract

**Background:**

Game-based cognitive assessments (GBCAs) have the potential to transform the field of cognitive testing by enabling more effective screening of age-related cognitive decline. However, we lack a strong understanding of the usability and overall user experience of these games. This is a risk because the primary target users for GBCAs, older people, are seldom involved in game design research and development.

**Objective:**

This study aims to address this gap by investigating the usability, acceptability, and enjoyability of GBCAs for older people.

**Methods:**

This study followed established practices for undertaking evidence-based systematic reviews.

**Results:**

The initial database search returned 15,232 records. After a thorough screening process, 8 studies remained for extraction and analysis. A synthesis of the included papers identified 2 overlapping yet distinct areas of focus: system usability and subjective user experience. Usability scores were mostly positive across the studies included. However, in several of the game studies, older adults and those with cognitive impairment tended to find GBCAs less usable. This trend was observed even when the games were explicitly designed for these populations, and the tasks were simplistic and representative of basic daily activities. In our second focus area, user experience, we identified the importance of perceived challenge in mediating gameplay experience across groups. That is, generating the appropriate level of difficulty for each user is important for positive user experiences, specifically enjoyment.

**Conclusions:**

On the basis of these findings, we identified key learnings for researchers interested in designing and developing GBCAs. These include (1) recognizing that validity is essential but not sufficient on its own; (2) clearly defining the intended user; (3) designing games that align with the unique preferences and needs of older people; and (4), whenever possible, providing each user with their optimal level of challenge.

**Trial Registration:**

PROSPERO CRD42023433298; https://www.crd.york.ac.uk/PROSPERO/view/CRD42023433298

## Introduction

### Background

In recent years, there has been growing enthusiasm about the use of serious game–based technology. The primary objective of these games is to increase user engagement and motivation, thereby offering people a more pleasant environment to complete “serious” objectives that go beyond entertainment [1]. One area identified as being particularly well suited to the application of serious game technology is the field of cognitive assessment [2].

Serious games for cognitive assessment have typically been developed in 1 of 2 ways [3]. The first is “gamification,” the process of building game-like features onto a cognitive task. This includes adding points, time limits, or appealing graphics to the foundational design of a traditional task. The second is to create or use an “actual” game, where the entire environment has been purposefully designed to be a game. This approach, more commonly associated with the term *serious game*, offers more creative development options, player choice, in-game competition, overarching narratives, and integrated or blended design elements. This approach is commonly based on theories of cognition or reimagining commercial games rather than reproducing a traditional cognitive task [3]. However, there remains significant confusion and inconsistency across the literature regarding what constitutes a serious game versus a gamified task. Therefore, unless otherwise noted, this paper refers to both approaches collectively as game-based cognitive assessments (GBCAs).

### Traditional Cognitive Screening and the Case for Change

Traditional cognitive assessments are used by health professionals to evaluate a person’s cognitive, or “thinking,” functions to identify cognitive impairments or age-related declines, such as Alzheimer's disease (AD) [4,5]. Most clinicians initially use short cognitive screening tests (eg, 10-15 min) when assessing these functions. Although there are clear and well-evidenced benefits of using traditional cognitive assessment and screening tools (eg, the Montreal Cognitive Assessment has been shown to have high sensitivity [6]), there are also several issues that reduce their overall effectiveness. One concern often cited by users is that traditional screening tests can be boring and repetitive, thereby reducing user motivation and engagement during assessment. This is problematic because data obtained from individuals who are not motivated to perform a task to the best of their abilities may not be representative of their optimal performance, and this can cause misleading performance data (eg, false positives) [1]. Brief assessment tools also lack ecologic validity, meaning tasks may not translate to real-life circumstances [1,3,7]. In addition, assessments can sometimes create feelings of shame and distress [8]. Consequently, some people report significant test anxiety as well as self-stigma related to low literacy or education levels when completing cognitive tests [9,10]. Another limitation is the concerning association between cognitive performance and education level found with many screening tools [5]. Finally, some widely used neuropsychological tests are not culturally appropriate [11].

An initial attempt to address some of the concerns with traditional screening methods included digital software suites, such as CogTest and the Cambridge Neuropsychological Test Automated Battery [12]. Although these tools enable more consistent assessment and data capture, they are essentially digitized versions of traditional cognitive tests and, thus, are not gamified. Tests of this nature have encountered validation issues [12] as well as similar problems to traditional testing, such as a lack of user motivation when performing somewhat uninteresting tasks on a computer [2].

### GBCAs Offer Exciting Alternatives, but They Must Be Valid, Acceptable, and Motivating

Games have recently been seen as a viable alternative to address many of these issues and improve both testing engagement and validity [1,2,12]. The benefits of using GBCAs include increased participant motivation and engagement relative to traditional tasks [2,3]. GBCAs can also offer shorter, more cost-effective, and scalable assessments [13,14]. Unlike traditional “pen-and-paper” assessments, games do not need to be initiated by a care provider and can be designed for self-administration or delivery by nonclinicians [1,14]. In addition, they may support more ecologically valid assessment through realistic context and gameplay, thereby engaging cognitive processes in ways that closely mirror real-life situations [15,16]. GBCAs can also enable consistent tracking of an individual’s cognitive performance data to support the detection of subtle changes or variations in cognitive processes over time [14]. The richness of this type of data can provide invaluable population-level insight into subtle aggregate patterns, changes, or important impacts (eg, effects of exercise or smoking) on cognitive health across groups. This is an especially attractive prospect, given the increasing sophistication of machine learning models.

However, to justify their use, GBCAs must function as effective and reliable cognitive assessment tools (ie, psychometrically validated) while also being usable, acceptable, and motivating. Much of the current literature on GBCAs assumes that the simple process of gamification improves user engagement and creates a more satisfying and less tedious experience than traditional pen-and-paper or digitized neurological tests. Although many games have been found to achieve this [1-3], this is not always the case. For instance, Birk et al [17] found that adding game elements to standard cognitive tests (eg, go/no go; n-back tasks) did not improve user engagement when compared with the original task. Gamification actually reduced test performance for the go/no go task. Tong et al [18] reported difficulties in testing a carnival whack-a-mole game with older users, also based on the go/no go paradigm, because of ergonomic issues with mobile and touchscreen devices (eg, the difficulty holding a touch device). It is also important to recognize that there may be restrictive settings or social contexts where GBCAs may be impractical, and traditional approaches may be more appropriate.

Overall, when games are not appropriately designed for their target users or contexts, they risk being neither acceptable nor motivating. Similarly, games might be skillfully designed and psychometrically validated but not adequately evaluated from users’ perspectives. A systematic review of game-based interventions for neuropsychological assessment, training, and rehabilitation found that user experience evaluation was performed in <25% of interventions, with usability and enjoyability assessed in only 13% and 18%, respectively [19]. For a technology specifically aimed at increasing user engagement and enjoyment, this lack of user design input and evaluation appears to be a barrier to achieving the technology’s intended outcomes.

### GBCAs for Older Users

The group of interest for this study is older people (aged ≥50 years) who have or may be at risk of cognitive decline and, consequently, neurocognitive disorders (eg, dementia, such as AD). Recent research on aging has suggested that groups considered marginalized (eg, people experiencing homelessness or incarceration) are at risk of age-related conditions, such as frailty and cognitive impairment [20,21], once they reach the age of 50. Thus, to ensure adequate inclusion of people considered marginalized who are at risk of cognitive decline, we define older people as those aged ≥50 years.

Concerns about user engagement are exacerbated for game assessments aimed at detecting cognitive decline such as dementia because the target group for such tests is typically older people. This represents a challenge because games are rarely designed for or tested with older people. By contrast, there is promising—but preliminary—research to suggest that people who either have or are at risk of cognitive decline can play, attend to, and enjoy digital games, even when they do not have previous experience with touchscreen devices [22]. However, their ability to successfully complete gamified tasks appears to depend on the specific application and mechanics used within a game [22]. More broadly, the game preferences of older people seem to be different from those of younger cohorts, with studies reporting the preferences of older users gravitating toward intellectually stimulating games (ie, puzzle, educational, and strategy games) [23] that enable them to compete for high scores, require only a single player, and emphasize intellectual challenge over quick reflexes [22]. According to Blocker et al [24], games with violent content, fantasy characteristics, or interactive web-based components are not preferred by older users. A qualitative study investigating the cognitive game preferences of older people in prison reported similar findings [25], with participants suggesting a need to avoid “childish” game design, which some found condescending.

### The Useful Concepts of Usability and User Experience

Across the design literature, usability and user experience are cited as major determinants of the successful adoption of any information system. Therefore, both are useful concepts when considering the acceptability, motivation, and engagement generated by any GBCA. Usability is the capacity of an object (ie, a digital game-based application) to functionally serve its intended purpose (ie, valid cognitive assessment) through qualities ranging from technical efficiency to conformity and configuration to ease of use [26]. According to the International Organization for Standardization (ISO: 9241-210:2019) [27], this refers to “the extent to which a system, product or service can be used by specified users to achieve specified goals with effectiveness, efficiency, and satisfaction in a specified context of use.” According to Hassenzahl and Tractinsky [28], usable systems or products enable users to achieve pragmatic objectives successfully and efficiently without obvious barriers.

On the other hand, user experience refers to the feelings a person has while interacting with a product under particular conditions [26]. This means that it goes beyond meeting the instrumental (pragmatic) need for usefulness and efficiency that good usability provides. User experience includes the need to achieve hedonistic goals [28,29], which involve “users’ emotions, beliefs, preferences, perceptions, comfort, behaviors, and accomplishments that occur before, during and after use” [27]. Although there is a clear overlap, the distinction between the concepts is that good usability makes it easy for someone to use something, whereas good user experience makes using that thing feel enjoyable or satisfying.

In the context of gamified technology, usability is focused on technical features such as a seamless interface, ergonomics, clear instructions, minimal in-game bugs or errors, and other features that facilitate use, whereas user experience is concerned with impacting a person’s subjective experience about the contents of a game, that is, a compelling narrative, an exciting challenge, motivation toward certain objectives, and so on [26]. There are more detailed and stratified definitions of usability and user experience across the user design literature (eg, refer to the 10 Usability Heuristics for User Interface Design by Nielsen [30]). However, consistent with our simplified definition of GBCAs, we intentionally use the basic demarcations of usability and user experience defined earlier to enable a consumable preliminary synthesis and analysis of this still-developing area of inquiry (ie, user evaluations of GBCAs for older people).

Arguably, the critical success factor for any GBCA is a coupling of user evaluation and psychometric validity. This is particularly true for older people at risk of, or presenting with, cognitive decline who are likely to have different preferences and experiences from other cohorts. However, much of the current literature is skewed toward investigating psychometric validity before or without evaluating usability and user experience. For instance, although several reviews in recent years have investigated GBCAs [2,3,31-34] and some have reported summary results on user evaluation methods (ie, how many studies conducted user evaluation) [19,35], there remains a limited understanding of the importance of user evaluation findings. This appears to be a risk, as even the most clinically useful game in the world is unlikely to succeed at scale if it is neither functional nor engaging.

### The Aims of This Study

This systematic review attempts to address some of these concerns by investigating the usability, acceptability, and enjoyability of GBCAs that have undergone psychometric validation. The intention is to better understand the nexus of clinical usefulness, usability, and user experience and, in turn, report preliminary lessons learned from user evaluations of validated GBCAs.

## Methods

This study (protocol registered on PROSPERO [CRD42023433298]) followed established practices for undertaking evidence-based systematic reviews, including the PRISMA (Preferred Reporting Items for Systematic Reviews and Meta-Analyses) checklist (Multimedia Appendix 1) and the Scottish Intercollegiate Guidelines Network methodology checklist for study quality appraisal (Multimedia Appendix 2 [16,36-43]).

### Data Collection, Extraction, and Quality Assessment

Independent and systematic database searches of PsycINFO, Embase, IEEE, and MEDLINE were conducted by the first author (RM) based on the predefined search terms summarized in Table 1. Broad search parameters were set, given the disparity of terminology used across the GBCA research. Relevant studies were then imported into Covidence, ready for initial screening. Title screening was manually completed by RM, where obvious exclusions and duplicates were removed. Duplicates were removed automatically via Covidence’s duplicate removal function. This was followed by independent and systematic screening of relevant abstracts by 2 reviewers (RM and MD), who applied predefined inclusion and exclusion criteria to assess all abstracts.

**Table 1:** Database search terms. Note: the truncation symbol * (asterisk) searches for multiple variants of a word (eg, singular, plural, different conjugations, etc) all at once (eg, cogn* includes searches for cognition, cognitive, cognitively, cognizant, etc). MCI: mild cognitive impairment; VR: virtual reality.

Topic area	Search terms
Serious games	game* OR gami* OR virtual reality OR VR AND
Cognitive	cogn* OR MCI OR dementia OR neurocog* OR neuropsych* AND
Assessment	assess* OR test* OR measur* OR collect* OR detect*

Textbox 1 presents the inclusion and exclusion criteria. Important definitions and justifications relating to the inclusion and exclusion criteria are discussed more thoroughly in the Discussion of important terms subsection. When disagreements arose at the abstract screening level, the second author (YIJH) facilitated a resolution through a 3-way vote. Full-text screening was then conducted by RM and MD. Conflicts at this stage were again handled through 3-way voting consensus involving YIJH, RM, and MD. At the full-text screening stage, there were disagreements with 12 (15%) of 83 papers, which necessitated additional consensus voting. Finally, a thorough data extraction process was conducted by RM, including a risk of bias assessment. We used the Scottish Intercollegiate Guidelines Network “Methodology Checklist 4: Case Control Studies” [36] guidance to assess the risk of bias for each included study. A detailed risk of bias assessment can be found in Multimedia Appendix 2. This final extraction process was reviewed by all authors to ensure consensus and quality control.

**Inclusion and exclusion criteria.**

**Inclusion criteria**

- User evaluated
- People aged ≥50 years
- Psychometrically validated
- Assessment or screening
- Cognitive functioning
- Primary research
- 2015 onward
- Digital serious games and gamification of task

**Exclusion criteria**

- Did not include target population or outcome
- Intervention with high immersion (via head-mounted display, etc)
- Training or rehabilitation focused without assessment
- No psychometric validation
- No user evaluation
- No data collection performed (ie, game performance data not collected)
- Inappropriate study design (reviews, conference abstracts, etc)
- Focus on social cognition only

### Discussion of Important Terms

This section summarizes and justifies several key concepts relevant to our inclusion criteria in Textbox 1, as well as our overall search strategy. It is important to emphasize that (1) our search strategy was intentionally broad to minimize the risk of missing any relevant GBCA studies and (2) our inclusion criteria were intentionally narrow to address our research aim of investigating the usability, acceptability, and enjoyability of GBCAs that have undergone psychometric validation.

#### User-Evaluated Study

The key criterion in this review was that a GBCA must have been user evaluated through primary research. This required the explicit inclusion of methods to measure usability, user experience, user motivation, user preference, enjoyment, or acceptability of the game.

#### GBCA Overview

Our definition of GBCA mirrors the broad definition included in the Introduction section. That is, both simple task gamification and more “from scratch” serious games were included. In addition, our focus was technology-based GBCAs; thus, we only included digital GBCAs (ie, games that could be played on computers, tablets, phones, etc).

#### Target Group

The target group for this review was older people (aged ≥50 years) who had, or were at risk of, cognitive decline and, consequently, neurocognitive disorders (eg, dementia, such as AD). Although there are numerous GBCAs that have been designed and tested to detect cognitive impairment associated with other disorders across the life course (eg, attention-deficit/hyperactivity disorder, depression, traumatic brain injury, drug- and alcohol-related cognitive deficits, etc), this paper is interested specifically in the assessment of cognitive decline in older people. This demarcation enables a manageable and specific grouping of findings.

#### Validated Study

To be included in this review, a GBCA must have been psychometrically validated with a sample of older users (aged ≥50 years). This validation could have occurred in the same study as the user evaluation or in a previous validation of the same game. In other words, evidence of validation was a prerequisite for inclusion in this review, but validation of the game was not necessarily required within the actual studies included in this review. According to previous definitions [3], validation included comparisons against a traditional cognitive screening tool (eg, Montreal Cognitive Assessment), a comprehensive neuropsychological battery, or a previous clinical diagnosis of cognitive impairment.

#### Cognitive Domains Included

A broad definition of cognitive functioning was applied for this study. Cognition refers to the brain’s ability to perceive, assimilate, organize, store, and manipulate information [44]. As such, cognitive functioning is an umbrella term that encompasses a range of integrated skills and domains that allow us to process and respond to information within our environment [44]. These domains include (but are not limited to) basic functioning (eg, processing speed and attention) and memory (eg, episodic memory, semantic memory, and prospective memory), visuospatial functions (eg, color perception and mental rotation), and executive functions (eg, planning, and decision-making). Relevant GBCAs testing any (or all) of these domains were included in this review, with the notable exception of social cognition. The clinical assessment of social cognition and decline in older people is not as well established as these other cognitive domains and was therefore intentionally excluded. However, investigating social cognitive domains should be seen as a critical consideration for future research.

#### Low-Immersion Interventions

This review made a distinction between low- and high-immersion games when deciding if a study should be included. Games that were deemed highly immersive from a technological perspective, such as those requiring headset technology and immersive 3D environments, were excluded. This review focused on GBCAs in a digital format that enabled accessibility through touch or mouse click capability. The main reason for this distinction was to ensure uniformity in findings. Although highly immersive games have significant potential benefits with respect to ecologic validity and integrated assessment of multiple cognitive functions [45], they have distinctive technology requirements when compared to computer or tablet-based game systems. In addition, factors such as motion sickness [46], which can affect enjoyability, are specific to virtual 3D headset environments and would have added unwanted complexity to this synthesis. Importantly, due to the disparity in the definition of serious games, gamification, and virtual reality across the literature, some interventions have been included in this review even when the authors refer to their interventions as *3D* or *virtual reality* game applications. However, these interventions aligned with our definition of a tablet-based and low-immersion GBCA and were thus included.

#### 2015 Onward

There is limited literature before 2015 that investigated games that were both psychometrically validated and user evaluated. There are 2 notable reviews that looked at validity before 2015 [2,34], but these reviews concluded that little work had been done in user evaluation of GBCAs before this time. In addition, a preliminary scan of literature before 2015 by the first author confirmed a lack of usability data from psychometrically validated games. Given our very broad search strategy, the significant growth of literature on GBCAs in the last 5 to 7 years, and a lack of user evaluation data before this time, a cut-off of 2015 for selection appeared justified.

## Results

### Overview

**Figure 1:**
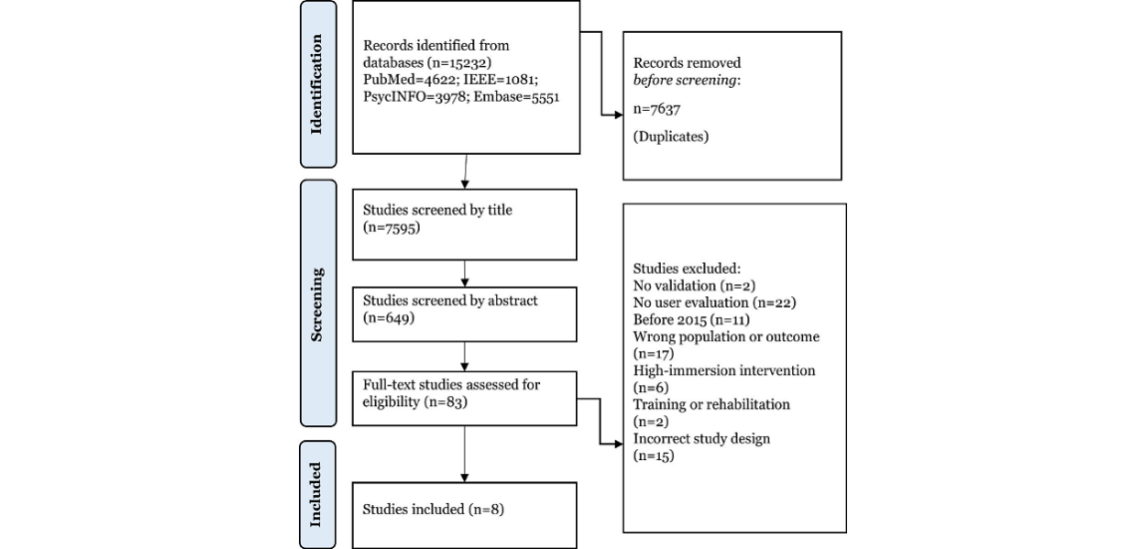
PRISMA flow diagram.

The initial database search on December 9, 2022, identified 15,232 records. There were 7595 articles left after removing duplicates. A total of 6946 articles were removed during the title screening for obvious exclusions, leaving 649 articles for abstract screening. Full texts were retrieved for 83 articles, of which 8 studies remained for extraction and analysis. More detail is provided in the PRISMA flow diagram in Figure 1, including reasons for exclusion at the full-text phase. The key study characteristics for all included papers are presented in Table 2. A summary of key psychometric validation results can be found in Multimedia Appendix 3 [15,47,48]. All studies included psychometric validity testing as part of their analysis except the Virtual Supermarket Test (VST) [37], where validity testing had been completed previously [15,47,48]. All games were designed and developed by the researchers involved in the included papers, with the exception of the Smart Aging Serious Game (SASG) [38], which was developed by Zucchella et al [49]. None of the included papers involved testing commercially available games; however, 2 of the games [39,40] were experimentally controlled versions of existing games. In addition, 4 of the 8 game studies were noted by the authors as being “virtual reality” interventions, which attempted to replicate real-life activities to assess cognition. However, all these games had low immersion and adhered to our definition of a digital GBCA (refer to the Discussion of important terms subsection for further justification).

**Table 2:** Summary of key study characteristics.

Studies	Game	Sample, n	Age, mean	Female (%)	Study aim
Cabinio et al [38], 2020	SASG^a^	Total: 139; controls: 107; MCI^b^: 32	Controls: 76.47; MCI: 76.75	Controls: 49.5; MCI: 46.8	Tested the usability of SASG in a cohort of participants with MCI and determined the validity of the SASG in discriminating between a preclinical population with MCI and healthy controls.
Chesham et al [40], 2019	SMT^c^	Total: 52; young (18-35 y): 28; older (65-85 y): 13; oldest (≥85 y): 11	Young: 21.68; older: 70.54; oldest: 89.27	Young: 71.4; older: 53.9; oldest: 81.8	Examined the initial validity and usability of an experimentally controlled version of a popular TMM3 puzzle video game—the SMT.
Manera et al [41], 2015	Kitchen and Cooking	Total: 21; MCI: 9; AD^d^: 12	MCI: 75.8; AD: 80.3	MCI: 77.8; AD: 86.7	Conducted a feasibility study of patients with MCI and AD and related disorders using the game “Kitchen and Cooking.”
Nef et al [39], 2020	Numberlink puzzle task	Total: 55; young (18-31 y): 18; older (64-79 y): 14; oldest (86-98 y): 14; PD^e^: 4; HD^f^: 5	Young:21.83; older: 71.4; oldest: 89.4; PD: 67.5; HD: 49.4	Young: 66.7; older: 57.1; oldest: 85.7; PD: 50; HD: 50	Evaluated the feasibility and preliminary validity of a maze-like NL puzzle video game as a tool to assess cognitive and motor differences in adults and patients with neurodegenerative disorders.
Valladares-Rodriguez et al [16], 2017	Episodix	Total: 16; controls: 8; MCI: 3; AD: 5	Controls: 68.3; MCI: 75.8; AD: 75	Total: 75	Investigated if a video game could be designed and developed to assess episodic memory and predict early cognitive impairments in an ecologic setting with low immersion.
Vallejo et al [42], 2017	Virtual games	Total: 38; controls: 20; AD: 18	Controls: 74.6; AD: 77.8	Controls: 40; AD: 50	Evaluated the usability and the screening potential of several low-immersion virtual game tasks for patients with AD.
Wang et al [43], 2022	GBCA^g^	Total: 124; controls: 57; NCDs^h^: 67	Controls: 72.31; NCDs: 74.78	Controls: 69; NCDs: 63	Developed a game-based tool and evaluated its validity as an early screening for patients with cognitive impairment.
Zygouris et al [37], 2022	VST^i^	Total: 57; SCD^j^: 24; MCI: 33	SCD: 65.58; MCI: 68.39	SCD: 79; MCI:70	Assessed the VST’s usability in a sample of older adults with MCI and SCD. The paper analyzed how usability is affected by age, education, diagnosis, in-game performance, and familiarity with touch devices.

^a^SASG: Smart Aging Serious Game.

^b^MCI: mild cognitive impairment.

^c^SMT: search and match task.

^d^AD: Alzheimer's disease.

^e^PD: Parkinson disease.

^f^HD: Huntington disease.

^g^GBCA: game-based cognitive assessment.

^h^NCD: neurocognitive disorder.

^i^VST: Virtual Supermarket Test.

^j^SCD: subjective cog decline.

### User Evaluation Findings

#### Overview

Preliminary analysis of the included papers identified 2 distinct areas of focus: usability and user experience. As such, our proceeding synthesis is demarcated based on usability and user experience (applying the simple definitions of these constructs provided in the Introduction section). Reporting findings from these 2 overlapping yet distinctive areas provides a holistic perspective of user evaluation by incorporating both system-level usability results as well as more individualized user experiences of serious gameplay. A detailed overview of relevant study findings and key statistical results is presented in Multimedia Appendix 4 [50-56].

#### GBCA Usability

A total of 5 studies [37-40,42] included system-level usability results. All these studies involved participant self-report measures but varied in the level of detail participants were asked to report. Overall, the average usability, from a systems perspective, of the games presented ranged from good to excellent. Familiarity with computers or touch screens did not appear to have a significant effect on system usability for computers or touchscreen devices, which is a positive finding. However, there were distinct differences in system usability across age groups and cognitive functioning levels in some studies. An interesting relationship was also found between usability and game performance in the VST study [37]. These are discussed further in the subsequent sections.

In the SASG study [38], participants were simply asked to respond to some questions about their familiarity with computers and touch screens. Results showed that familiarity with computer systems did not influence the SASG game performance score. Although this is not in itself an assessment of usability, it revealed an important usability finding: a lack of digital experience did not impact overall cognitive performance scores in a sample of older game users with low digital literacy and experience. A potential reason for this result may be the cautious and simplistic design of the SASG interface, which supported usability for the target cohort. The game was built specifically for older and nonexpert users, and according to the authors, it did not require skilled digital abilities. Using a low-immersion, first-person perspective, the game required users to complete gamified versions of everyday tasks, such as watering flowers with the radio on and dialing a phone number. Furthermore, the authors suggested a key factor in the usability of their serious game intervention was the decision to use a touch screen instead of a mouse.

The remaining 4 studies used the system usability scale (SUS) to measure and analyze usability [57]. The SUS is a brief questionnaire (10 questions) using a 5-point Likert scale with a possible aggregate score ranging from 0 to 100. The SUS includes questions such as “I thought the system was easy to use” and “I needed to learn a lot of things before I could get going with this system.” According to previous studies [58,59], the average SUS score (50th percentile) is 68, a good score is between 71 and 84, and an excellent usability score is ≥85.

In the VST study [37], the average SUS score was 83.11 (SD14.6). No statistically significant differences were found between the participant groups, people with subjective cognitive impairment and mild cognitive impairment (MCI), regarding SUS scores. However, there was a significant correlation (*r*=−0.496; *P*<.001) found between the SUS score and the average time needed to complete the VST test trials, independent of the participant group. This suggested a relationship between game completion and usability: the lower the participants rated the game’s usability, the longer they took to complete it. Although this was an interesting finding, the direction of causality is unknown. As the authors noted [37], “It is unclear whether participants who took longer to complete the VST test trials provided a lower usability score as they were frustrated by the long time it took them to complete the exercise or if there are underlying usability issues that affected their performance thus resulting in a longer time to complete the VST test trials.” It is also worth noting that in the VST study, participants were asked to complete the VST exercise on their own. The examiner prepared the tablet and launched the VST game but did not assist the participants. In fact, researchers intentionally provided little support to participants once gameplay started. The reason for this was to assess the usability of the system in an environment where a user may need to self-administer the game without support.

In the Numberlink (NL) puzzle task [39], the SUS average was 93.38 (SD 5.72) for young adults, 93.33 (SD 6.25) for older adults, 83.39 (SD 11.79) for the oldest adults, 81.88 (SD 9.66) for people with PD, and 83.12 (SD 10.68) for those with HD. These scores reflected a statistically significant difference in usability ratings between groups (*P*=.02). There were similar findings in the search and match task (SMT) study, albeit with lower reported usability overall [40]. The average SUS score was 88.67 (SD 7.28) for younger adults, 79.09 (SD 15.50) for older adults, and 68.25 (SD 18.78) for the oldest adults. This indicated a significant effect of age group (*P*=.01), with usability ratings decreasing with increasing participant age. Finally, the virtual game study [42] reported a mean SUS score of 83.5 (SD 11.16) for the control group and 83.75 (SD 9.82) for people with AD. As such, similar to the VST study and contrary to the NL puzzle task and SMT studies, there was no significant group difference found in usability. There was also no significant difference in SUS scores found between participants with and without touch device familiarity.

#### GBCA User Experience

Five studies [16,39-41,43] examined user experiences of gameplay, specifically focusing on participant engagement with game elements, perceived motivation, and enjoyment. Overall, all the games presented were well received by users regarding motivation, enjoyability, and satisfaction. However, there were some important differences across the study findings. For instance, some studies identified a relationship between being cognitively healthy and improved user experience [40,43], whereas an opposite relationship was found in another study [41]. Furthermore, 2 studies identified the role of a health professional as essential to the success of their respective game interventions [16,41], which contrasted with the intent in studies to test implementing games in a self-administering environment [37,41].

In the Episodix study [16], a user experience questionnaire based on game usefulness and user motivation was completed by participants. The questionnaire was completed twice, once before and once after gameplay. Before playing the game, most participants reported low interest, motivation, and perceived usefulness of serious games; however, their experiences playing Episodix changed these perceptions. For instance, the average participant’s motivation to play video games increased by 40% after gameplay. Participants also indicated that the Episodix game seemed more engaging than the California Verbal Learning Test (CVLT) pen-and-paper test, which the game was based on. One potential reason for the game seeming more engaging than the CVLT may have been the comprehensive user-centered design process used in developing the game. A combination of validity testing, expert input, and user focus groups underpinned the development process. Through the design process, participants from both focus groups and the pilot experiment indicated a preference for a touch interface rather than a traditional keyboard and mouse setup. This finding was similar to that of the SASG game study. Technical developments were made to facilitate these preferences. The Episodix study [16] also reported important qualitative findings from user focus groups. The game was perceived as very useful by users, but they preferred it to be administered by a health professional rather than self-administered, regardless of whether they were confident interacting with the game on their own. Participants agreed that if both instruments (the CVLT and the game) were demonstrated to perform the same cognitive assessment, they would prefer to play the game. However, participants preferred the digital intervention to be referred to as a test because they perceived the term *video game* to have negative and nonserious connotations.

Both the NL puzzle task and SMT studies [39,40] assessed user experience through the Perception of Game Training Questionnaire [50]. Participants rated the extent to which they found playing the games enjoyable, challenging, frustrating, and motivating. Both games were developed based on experimentally controlled versions of commercially available puzzle games. In addition, both games offered different difficulty levels to create an appropriate level of challenge for a broad range of users. In the NL puzzle task study, ratings of enjoyment (*P*=.43), challenge (*P*=.07), frustration (*P*=.06), and motivation (*P*=.67) for the game did not differ significantly between groups. The users generally rated the game experience positively in both the healthy aging group and the group with cognitive impairment. However, there was a slight, albeit statistically nonsignificant, increase in rating the game session as challenging and frustrating among older participants and/or those with impairment, particularly participants with PD and HD. According to the authors, further adapting the difficulty levels to a person’s performance might partially reverse this trend. In the SMT study, there were significant differences between the 3 age groups regarding ratings of challenge (*P*<.001). Overall, young adults perceived the SMT as significantly less challenging than older adults (*P*=.02). There were also significant age group differences in average difficulty ratings for both the short version played by the young, older, and oldest adults (*P*<.001) and the long version (*P*<.001) played by the young and older adults. Despite this, there were no significant group differences regarding enjoyment, frustration, and motivation while playing the SMT task. One possible reason why challenge and difficulty levels varied across groups while enjoyment and motivation levels remained constant is that the SMT provided a total of 71 possible difficulty levels. This means that the game could be responsive to a user’s gameplay and adjust the level of perceived challenge accordingly.

In the GBCA study [43], the user experience questionnaire included questions on game ease-of-use (not technically user experience), whether users felt the game stories were interesting and familiar, users’ reactions to the interface design (typesetting, color, instruction, etc), and questions aimed at comparing the GBCA intervention with other cognitive assessment tools. The responses of the control group suggested that they were very satisfied with the game. By contrast, the average responses of participants with neurocognitive disorders were significantly lower than those of controls. Interestingly, lower scores given by the participants on the user experience questionnaire corresponded to a higher clinical dementia rating, older age, and a lower educational level. Higher levels of cognitive impairment appeared to adversely affect the participants’ ability to use the tablet to complete the GBCA intervention, as indicated by the poor ratings given to questions regarding the design of the GBCA intervention. This finding raises questions about the functional usability of the game as well as the more subjective user experience. Furthermore, older age had a negative influence on the participants’ satisfaction regarding the pictures, text, and story used in the game. This was somewhat surprising, given that the user interface of the GBCA intervention was intentionally designed to be acceptable for older users and included large pictures and buttons as well as spoken instructions so users could operate the app easily. The game could also be played by tapping buttons, drawing by dragging one’s fingers over the touch screen, and speaking into a microphone (using low-cost speech recognition technology).

The Kitchen and Cooking study [41] also captured user experiences. The participants completed a questionnaire about the game experience at 2 points during the study. Specifically, satisfaction, interest, motivation (intrinsic and extrinsic), emotional experience of gameplay, and fatigue levels were assessed. The overall results of the self-report questionnaire showed that the participants were highly satisfied, interested, and motivated by the game experience. Intrinsic motivation was significantly higher than extrinsic motivation. Average user experience scores did not change between the 2 sessions, thus confirming the overall positive evaluation of the game after repeated gameplay. However, it is worth noting that scores did not trend upward after the second session regarding motivation, as observed in the Episodix study [16]. Of particular interest in this study is that participants with AD reported being significantly more satisfied with the game than participants with MCI (*P*=.04). This contrasts with the findings from the NL puzzle task, SMT, and GBCA studies, where the inverse trend appeared. Finally, the authors suggested that a critical factor in the success of the intervention was the presence of a clinician. The game was designed as a tool to help patients train outside clinical consultations. However, the periodic supervision of the clinician helped to explain the functioning of the game to the patients and their families, keep track of the evolution of the performance, adapt the intervention to the patients’ changing needs, and maintain user motivation.

## Discussion

### Overview

The aim of this review was to investigate studies that evaluated the experiences of older people using GBCAs. Although the combined findings are from only 8 included papers, they establish promising preliminary user evaluation results for the use of GBCAs, albeit with some interesting implications for future design and development practices. Our review provides a much-needed synthesis of user evaluation findings from GBCAs, which have undergone psychometric validity testing. As such, we offer new insights regarding the relationship between the validity, usability, and user experience of GBCAs. This is particularly important with respect to the experiences of our target study cohort, older people (aged ≥50 years), whose input is greatly needed yet seldom included in GBCA design and development.

The first area of focus in this review was system usability. Usability scores were mostly positive across the studies included. However, in some of the games presented, there were trends toward lower usability scores for participants with cognitive impairment. This was observed even when games were explicitly designed for older people and/or those with impairment, and tasks were simplistic and representative of basic daily activities, such as in the VST. In the VST, a game that has been validated in multiple contexts [15,47,48], no significant differences were found between the groups with subjective cognitive impairment and MCI in usability scores; however, participants who took longer to complete the test trials had lower self-reported usability. Usability challenges were further identified in the NL puzzle task and the SMT. Although both games had good usability scores on average, they both identified significant differences in usability ratings not only across cognitive health but also age range. On average, in both games, the older or more impaired a person was, the less usable they found the respective game system.

These collective findings on usability have some interesting implications. Traditionally, it has been assumed that system usability can be increased by improving the technical interactions a person has with a system, the intuitiveness of that system, and by reducing any bugs or errors that generate needless complexity within that system. For example, Joddrell and Astell [60] significantly improved the usability of solitaire for people with dementia (mean age 84.17, SD 8.35; range 66-102) by adding accessibility features such as additional card control options, clearer gameplay layout, and audio-visual feedback cues. However, this review highlights that while these technical and functional system improvements may be important, they are not necessarily sufficient to produce usable interventions for this target group of users. Individual variations in age, cognitive function, and gameplay performance may all impact functional usability, even when the system has been technically adapted to be suitable for older users and/or those with impairment.

The second focus area, regarding subjective user experience, identified the importance of perceived challenge in mediating gameplay experience across groups. In the GBCA study, the healthy control group was more satisfied with their experience of using the GBCA intervention than the group of participants with cognitive impairment, whereas the opposite trend appeared in the Kitchen and Cooking game. In the Kitchen and Cooking game, a simplistic interface based on a basic cooking task, game satisfaction was higher among the participants with AD than among participants with MCI. In the SMT task, there was a statistically significant difference in how challenging older and younger people found the game. However, levels of enjoyment and motivation remained the same. These contrasting results may be related to perceived gameplay challenges. For instance, the NL puzzle task, the SMT, and the GBCA were, on average, more complex than the Kitchen and Cooking game, with the NL puzzle task and SMT offering different levels of complexity for different users.

According to the broader literature on game design preferences, people want to be challenged to a level that will satisfy their need for competence [25]. Applying self-determination theory, it has been shown that a player will experience low competence if the challenges are too great (eg, game controls are overly complex or enemies are too numerous), and this will have a negative impact on a person’s ability to enjoy the game [61]. If a game is too difficult, it can become frustrating and discouraging, reducing a user’s motivation, engagement, and enjoyment. The optimal challenge (ie, the sweet spot) for any player is mediated by that individual’s threshold for difficulty [62,63]. This may explain why basic gamified versions of simple tasks, such as in the Kitchen and Cooking game, were less motivating or enjoyable for younger participants and those without cognitive impairment. It may also explain why more “from scratch” serious games that offered more difficulty levels, such as the NL puzzle task and SMT, had constantly high enjoyment and motivation scores across the younger, older, and oldest groups of users.

Another noteworthy finding in this review relates to the role of a health professional during game-based assessment. Two studies identified the role of a health professional as essential to the success of their respective game interventions. In the Episodix game, although users demonstrated improved appreciation for the value of GBCAs after gameplay, this increased appreciation appeared to stem from the value of the game as a health tool. The presence of a clinician seemed to underpin this, as the game was perceived as very useful by users, but they preferred it to be administered by a health professional rather than self-administered. Likewise, in the Kitchen and Cooking study, the authors suggested a critical success factor for the intervention was the presence of a clinician. These findings raise questions about self-administration, which is seen as one of the most exciting (and valuable) applications of serious game technology [15]. Self-administration of GBCAs has been recently identified as a way to overcome some of the issues with formal assessment [15], such as significant test anxiety as well as self-stigma regarding low literacy or education levels when performing cognitive tests [9,10]. Self-administration may also empower people to take control of their own health earlier and more proactively than standard approaches. However, the findings of this review suggest that this autonomy and control may come with some unintended consequences, such as a lack of confidence in undertaking assessments without support, as well as a lower perceived value or legitimacy of the assessment. This is important to recognize as a potential downside for self-administrated GBCAs.

### The Objectives of GBCAs and Subsequent Tensions With Usability and User Experience

One approach often taken when designing and developing cognitive games for older people and/or individuals with cognitive impairment is to make the game easy. This appears to have been the design strategy in games such as SASG, VST, and Kitchen and Cooking, where the games were based on simple daily living tasks, and the design and development of the games were tailored to people with cognitive decline. These games were quite successful at engaging older people with significant cognitive impairment. However, given that the objective of a GBCA is to assess maximal cognitive capacity, adapting the floor and ceiling to make games easier may not always be desirable. This is because the reason for adding complexity to a GBCA, besides increasing user engagement, is to improve the sensitivity of a game to detect cognitive functioning more accurately. In other words, a game that is too simple or too domain specific may not be relevant for healthier older adults or those with milder or more complex forms of cognitive impairment, even if it is more appropriate for people with more significant cognitive decline. For instance, the basic requirement of shopping for groceries in the VST may be suitable for people with moderate neurocognitive disorders; however, it may not detect subtle impairments or small cognitive changes over time in healthier participants. More simplistic games are also unlikely to be very enjoyable or challenging (ie, satisfying a need for competence) for many people without neurocognitive disorders (as was observed in some of the studies included). This reduces their viability as a scalable screening tool. By adding more challenge and complexity, it becomes feasible to engage a broader user group and assess multiple cognitive domains. This, of course, comes at the risk of marginalizing the most vulnerable and arguably important cohort: those with significant impairments. It is also important to note that people with more significant neurocognitive disorders are unlikely to be the intended target of most GBCAs, or even traditional cognitive screening. This is a tension that has been seldom discussed or investigated in the growing body of literature on GBCAs.

Overall, the goals of usability and user experience—foundational to human-centered design approaches—and the intention of GBCAs and cognitive testing, in general, reveal a potentially problematic misalignment that warrants further discussion. For instance, according to ISO, the basic requirement of usability is that a product can be used (by target users) to achieve specific goals with effectiveness, efficiency (ie, in minimum time and with minimum errors), and satisfaction in a specified context [27]. However, is it reasonable, desirable, or even possible to meet this standard when developing a GBCA? If the primary goal of a cognitive test is the detection of cognitive deficits, the implication is that people with cognitive impairment will likely (1) fail to achieve certain tasks, (2) take longer to complete objectives, and (3) make more errors than cognitively healthy people.

In fact, reliable detection of these “failures” is essential to ensure a valid and clinically meaningful test of cognitive performance. Thus, intentionally producing user failure states is a central design tenet when developing GBCAs, and this, as we have identified through this review, may cause reductions in usability and subjective user experiences.

However, while this may risk departing somewhat from the principles of user-centered design, it is important to recognize that the “serious” objective of a GBCA is not necessarily to maximize unconstrained usability and user experience; rather, it is to produce a cognitive assessment that is more motivating and engaging than a traditional task. As traditional tasks are sometimes reported to be tedious, boring, anxiety provoking, and stigmatizing, the objective of a GBCA is to improve the subjective user experience relative to traditional approaches while maintaining functional usability. This implies that a GBCA may not necessarily need to be maximally fun but simply “fun enough” while being motivating, engaging, and technically usable. Furthermore, what “usable” means in this context is potentially hard to reconcile with the ISO aim of effectiveness, efficiency, and satisfaction. For instance, if an intervention is too easy to use, can it adequately produce the failure states that are the primary objective of a GBCA?

These open questions become even more interesting when we consider that the intended application of GBCAs, at least in the near future, is likely to be a relatively short (eg, 10-20 min) user experience (ie, replacing a cognitive screen), which is only “played” by users sporadically. The implication is that traditional user-centered game design that focuses on intrinsically motivating features, such as narrative and social connectedness, may not be feasible or desirable. This, in many ways, is misaligned with the overarching goals of human-centered design, yet it is arguably more consistent with the preferences of older people taking a cognitive test. In fact, this was what we reported in a recent qualitative study investigating the GBCA preferences of older people in prison [25]. We found that GBCAs with numerous immersive game features risked being perceived as too childish for the serious context of a cognitive test. This was also likely the case in the Episodix study sample, where participants preferred the digital intervention to be referred to as a test because they perceived the term game to be nonserious [16]. However, we argue that these tensions do not reduce the value of incorporating usability and user experience principles into the development of GBCAs; rather, they simply reframe what the feasible intention of a GBCA ought to be in the context of user experience and usability. Perhaps “good enough” usability and user experience are good enough?

Our conclusion is that there is an inherent tension between usability, user experience, and GBCAs that (1) in the meantime should be carefully considered when designing and developing GBCAs, especially for older people; and (2) requires additional research to further explore and potentially resolve. We offer some key lessons in the Implications for Practice subsection. Regarding future research, we suggest an urgent need for further mixed methods research that incorporates the qualitative preferences of older people using GBCAs with rigorous validity testing. In addition, there is a need to explore what “good enough” usability and user experience may mean in this context and how these concepts can be tested and incorporated into game design, development, and improvement.

### Limitations

This review has some limitations. Many of the participant samples were small, and the studies involved slightly different target user groups. Most studies focused on user evaluation as a secondary outcome, with psychometric validity testing being the primary aim in most papers. All the games included in this review were distinct from one another regarding design and development processes and, as a result, the end products. Combined with the disparities in sampled participants, this heterogeneity makes it inappropriate to make specific recommendations regarding which precise techniques work for whom in what contexts. This is further complicated by the fact that most of the games did not seem to be publicly available, creating uncertainty about how well developed and sophisticated the games actually are in practice. This lack of access to the games, beyond what was described and visualized in the primary research papers, also inhibited a more comprehensive review of specific design choices based on a more technical analysis of mechanics and processes. However, despite these limitations, we were still able to interpret the findings in the context of the broader literature on serious games and provide general lessons learned for designing and developing GBCAs.

In addition, there were also some limitations related to the methods applied for user evaluation. Most user evaluation data were collected via self-report and are thus exposed to the well-established biases associated with this collection method. Furthermore, although the SUS is well validated, it is short and generic. Thus, it does not present a comprehensive assessment of game usability, and its validity for people with neurocognitive disorders has not been established. Other reported usability and user experience findings came from disparate tools, some of which were customized from other scales or created based on theory. This created additional barriers when attempting to synthesize the findings quantitatively and prevented a meta-analysis. Some of these limitations were evident in the quality appraisal, in which a case-control checklist was used to assess the risk of bias. This basic checklist was deemed appropriate because most of the study papers included games that were still in the pilot stage of development and, thus, not ready for more sophisticated diagnostic appraisal. Regardless, it is hoped that a more sophisticated and suitable appraisal tool can be used in future research to adequately assess the quality of papers investigating the psychometric validity and user experiences of GBCAs.

### Implications for Practice

#### Overview

This systematic review aimed to synthesize the user evaluation data from psychometrically validated GBCAs played by older people. As part of this process, we have identified key lessons for developing potentially transformative assessments using gamified technology. These preliminary key lessons have been developed based on our systematic review of the included studies, our own primary research experiences (eg, Mantell et al [25]), and a broader synthesis of relevant theory and literature (eg, self-determination theory). Given that this is still a relatively new area of scientific inquiry, we hope that these lessons can serve as guidance for clinicians, researchers, and game developers who find themselves navigating the complexity of creating GBCAs that are both clinically effective and offer “good enough” user experiences and usability. More broadly, we hope this review can support and inform more evidence-based and rigorous design, development, and evaluation of GBCAs for older users in the future.

#### Validity Is Very Necessary but Not Sufficient

A game may function as an assessment tool in theory, but if it is not usable or engaging, it is unlikely to work in practice. Input from both users and game designers, at the outset and throughout the design process, is the best way to ensure a game is suitable. This is usually best done alongside an expert reference group working to ensure the game has psychometric validity [13]. The more the target users, developers, and cognitive experts collaborate, the more likely the game will succeed. Furthermore, researchers should always plan to evaluate game usability, as well as user experiences and preferences, to formally investigate the success of their game (and identify any scope for improvement).

#### Clarify the Intended User

There is a complex relationship between cognitive decline, usability, and user experience as people age. Many older people with cognitive impairment will respond better to simple gamification of realistic tasks. More complex serious games risk becoming less appealing as people become older or more impaired, and this will impact usability and user experience. However, the more rudimentary the game-based task, the less likely it is to engage younger adults or those with better cognitive function. To proactively address this tension, developers should begin with a critical question: who is this intervention for, and what exactly is the game intended to assess? For instance, “good enough” usability and user experience are probably good enough for a short and sporadic cognitive screener targeting older adults at risk of impairment. However, this may not be the case for a more expansive game trying to also engage younger cohorts and track more subtle cognitive changes over time.

#### Create Games That Align With the Unique Preferences and Needs of Older People

Older people appear to prefer games that avoid childlike features, are intellectually focused (eg, puzzles), and/or are based on the replication of real-life tasks or challenges. Avoiding fantasy, violent content, and multiplayer interaction is recommended. Large digital touchscreen devices, such as tablets, also seem to be more usable and intuitive for older people than smaller devices, mouses, or virtual reality [16,38,64]. However, tablets can also create ergonomic difficulties for older people who may be frail or have specific neurological conditions (eg, difficulty holding the device steadily, dyspraxia, or visuospatial impairment), and these factors need to be accounted for [18].

#### Providing Each User With Their Optimal Challenge Is Key

Tailoring the level of difficulty (known as difficulty balancing) to an individual’s capacity should be attempted wherever possible in the design of GBCAs. It has been shown that this personalization does not need to be highly sophisticated to be useful in increasing user experience [37,39]. Introducing various levels or randomly generating different design elements or challenges per round can also make it possible to detect more subtle impairments and reduce learning effects [37]. In addition, it can also alleviate some of the tension between providing games that are suitable for older people or those with more impairment and maintaining sensitivity for more subtle cognitive decline, as more levels mean a lower floor and higher ceiling.

## Appendix

Multimedia Appendix 1 PRISMA checklist.

Multimedia Appendix 2 Risk of bias.

Multimedia Appendix 3 Validity summary.

Multimedia Appendix 4 User evaluation summary.

## Abbreviations

AD: Alzheimer's disease
CVLT: California Verbal Learning Test
GBCA: game-based cognitive assessment
ISO: International Organization for Standardization
MCI: mild cognitive impairment
NL: Numberlink
PRISMA: Preferred Reporting Items for Systematic Reviews and Meta-Analyses
SASG: Smart Aging Serious Game
SMT: search and match task
SUS: system usability scale
VST: Virtual Supermarket Test
