# Supplementary material for: Evaluating the User Experience and Usability of Game-Based Cognitive Assessments for Older People: Systematic Review
Source: JMIR Aging. 2025 Jun 11;8:e65252. doi: 10.2196/65252 (PMC12198696; doi:10.2196/65252)
Supplement: Multimedia Appendix 2 [file aging_v8i1e65252_app2.docx]

**Appendix 2: risk of bias appraisal**

Healthcare Improvement Scotland (Scottish Intercollegiate Guidelines Network (SIGN)): Methodology Checklist 4: Case Control Studies [36]

| AUTHOR(S) | GAME | INTERNAL VALIDITY | | | | | | | | | | | OVERALL ASSESSMENT | | |
| --- | --- | --- | --- | --- | --- | --- | --- | --- | --- | --- | --- | --- | --- | --- | --- |
|  |  | **1.1** | **1.2** | **1.3** | **1.4** | **1.5** | **1.6** | **1.7** | **1.8** | **1.9** | **1.10** | **1.11** | **2.1** | **2.2** | **2.3** |
| Cabinio [38] | Smart Aging Serious Game (SASG) | + | + | * | - | + | - | - | + | + | - | + | **(+)** | **Yes** | **Yes** |
| Chesham [40] | Search and Match Task (SMT) | **+** | * | **+** | **-** | **+** | **+** | **+** | **+** | **+** | **+** | **+** | **(++)** | **Yes** | **Yes** |
| Manera [41] | Kitchen and  Cooking | **+** | **+** | **+** | **+** | **+** | **+** | **+** | **-** | **+** | **-** | **-** | **(+)** | **-** | **Yes** |
| Nef [39] | NL Puzzle Task | **+** | ***** | **+** | **-** | **+** | **+** | **+** | **-** | **+** | **-** | **+** | **(++)** | **Yes** | **Yes** |
| Valladares-Rodriguez [16] | Episodix | **+** | **+** | **-** | **-** | **+** | **-** | **-** | **+** | **+** | **-** | **+** | **(+)** | **Yes** | **Yes** |
| Vallejo [42] | Virtual Games | **+** | **+** | **+** | **+** | **+** | **+** | **+** | **+** | **+** | **-** | **+** | **(++)** | **Yes** | **Yes** |
| Wang C [43] | Game-Based Cognitive Assessment (GBCA) | **+** | **+** | **+** | **+** | **+** | **+** | **+** | **+** | **+** | **-** | **+** | **(+)** | **Yes** | **Yes** |
| Zygouris [37] | Virtual Supermarket Test (VST) | **+** | **+** | **+** | **+** | **+** | **+** | **-** | **+** | **+** | **-** | **-** | **(++)** | **Yes** | **Yes** |

** these studies assessed game performance across age ranges, and thus it was not possible to sample from comparable populations with respect to age.*

**Section 1 – internal validity**

- 1. The study addresses an appropriate and clearly focused question.

*Selection of subjects*

- 1. The cases and controls are taken from comparable populations.
  2. The same exclusion criteria are used for both cases and controls.
  3. What percentage of each group (cases and controls) participated in the study?
  4. Comparison is made between participants and non-participants to establish their similarities or differences.
  5. Cases are clearly defined and differentiated from controls.
  6. It is clearly established that controls are non-cases.

*Assessment*

- 1. Measures will have been taken to prevent knowledge of primary exposure influencing case ascertainment.
  2. Exposure status is measured in a standard, valid and reliable way.

*Confounding*

- 1. The main potential confounders are identified and taken into account in the design and analysis.
  2. Confidence intervals are provided.

**Section 2 – overall assessment of the study**

- 1. How well was the study done to minimize the risk of bias or confounding?
  2. Taking into account clinical considerations, your evaluation of the methodology used, and the statistical power of the study, do you think there is clear evidence of an association between exposure and outcome?
  3. Are the results of this study directly applicable to the patient group targeted by this review?

**References**

16. Valladares-Rodriguez S, Perez-Rodriguez R, Facal D, Fernandez-Iglesias MJ, Anido-Rifon L, Mouriño-Garcia M. Design process and preliminary psychometric study of a video game to detect cognitive impairment in senior adults. PeerJ. 2017;5:e3508. [doi: 10.7717/peerj.3508] [Medline: 28674661]

36. Sleith C. Methodology checklist 4: case control studies healthcare improvement Scotland. Scottish Intercollegiate Guidelines Network. 2012. URL: https://www.sign.ac.uk/using-our-guidelines/methodology/checklists/ [accessed 2025-05-29]

37. Zygouris S, Segkouli S, Triantafyllidis A, Giakoumis D, Tsolaki M, Votis K, et al. Usability of the virtual supermarket test for older adults with and without cognitive impairment. J Alzheimers Dis Rep. May 02, 2022;6(1):229-234. [doi: 10.3233/adr-210064]

38. Cabinio M, Rossetto F, Isernia S, Saibene FL, Di Cesare M, Borgnis F, et al. The use of a virtual reality platform for the assessment of the memory decline and the hippocampal neural injury in subjects with mild cognitive impairment: the validity of smart aging serious game (SASG). J Clin Med. May 06, 2020;9(5):1355. [doi: 10.3390/jcm9051355] [Medline: 32384591]

39. Nef T, Chesham A, Schütz N, Botros AA, Vanbellingen T, Burgunder J, et al. Development and evaluation of maze-like puzzle games to assess cognitive and motor function in aging and neurodegenerative diseases. Front Aging Neurosci. Apr 21, 2020;12:87. [doi: 10.3389/fnagi.2020.00087] [Medline: 32372942]

40. Chesham A, Gerber SM, Schütz N, Saner H, Gutbrod K, Müri RM, et al. Search and match task: development of a Taskified match-3 puzzle game to assess and practice visual search. JMIR Serious Games. May 09, 2019;7(2):e13620. [doi: 10.2196/13620] [Medline: 31094325]

41. Manera V, Petit P, Derreumaux A, Orvieto I, Romagnoli M, Lyttle G, et al. 'Kitchen and cooking,' a serious game for mild cognitive impairment and Alzheimer's disease: a pilot study. Front Aging Neurosci. Mar 17, 2015;7:24. [doi: 10.3389/fnagi.2015.00024] [Medline: 25852542]

42. Vallejo V, Wyss P, Rampa L, Mitache AV, Müri RM, Mosimann UP, et al. Evaluation of a novel serious game based assessment tool for patients with Alzheimer's disease. PLoS One. May 4, 2017;12(5):e0175999. [doi: 10.1371/journal.pone.0175999] [Medline: 28472049]

43. Wang CS, Wu J, Hsu W, Chien P, Chen P, Huang Y, et al. Using self-administered game-based cognitive assessment to screen for degenerative dementia: a pilot study. J Alzheimers Dis. 2022;86(2):877-890. [doi: 10.3233/JAD-215142] [Medline: 35147533]
