# Supplementary material for: Evaluating the User Experience and Usability of Game-Based Cognitive Assessments for Older People: Systematic Review
Source: JMIR Aging. 2025 Jun 11;8:e65252. doi: 10.2196/65252 (PMC12198696; doi:10.2196/65252)
Supplement: Multimedia Appendix 3 [file aging_v8i1e65252_app3.docx]

**Appendix 3: validation summary**

| GAME | GAME TYPE | DOMAIN(S) TESTED | VALIDATION METHOD | VALIDATION RESULTS |
| --- | --- | --- | --- | --- |
| Episodix | Gamified tasks based on the California Verbal Learning Test (CVLT) | Episodic memory | - Comparison with traditional screening tool (MMSE), in order to gather gold standard data to correlate with Episodix's variables. - Comparison with test battery: California Verbal Learning Test (CVLT), Memory Alteration Test (MAT) and Informant Questionnaire on Cognitive Decline in the Elderly (IQCODE) - Statistical analysis: data collected from interactions with Episodix was analyzed using machine learning techniques. Namely, support vector machine, linear regression and random forest techniques were applied. Analysis focused on *predictive validity* to detect cognitive impairment and discriminate people with MCI, AD and healthy controls. | - Predictive and classification capabilities of Episodix (i.e. correctly detecting cognitive impairments): the ability to correctly classify individual subjects was highly satisfactory, especially when the one-left-out training/testing methodology (machine learning model assessment technique) was applied. - Convergent validity: the best correlations between CVLT and Episodix variables were failures, guesses and omissions during short delay recall phases with clues, as well as omissions from immediate recall phase during all trials of first and second walks. Moreover, time duration provided a good correlation with CVLT, particularly during short delay clued phase and free recall phase. |
| Game-Based Cognitive Assessment (GBCA) | 13 mini-games/tasks with an overarching game narrative (total score of 100) | Visuospatial Perception  Attention  Language  Memory  Executive Function | - Comparison with traditional screening tool (CDR, CASI, and MMSE) - Statistical analysis: The chi-square and independent t-test were used to *examine the differences* between people with neurocognitive disease and healthy controls with regards to demographic data, scores on the CASI, MMSE, and GBCA, and responses to the user questionnaire. *Pearson’s correlations* were also used to calculate the linear correlations between the MMSE, CASI, and GBCA, and their respective items. A *receiver operating characteristic (ROC)* curve was created to assess the overall performance of the CASI, MMSE and GBCA at discriminating between NCD subjects and HCs. | - Correlation analysis: for the HC group, age and education level were not significantly correlated with scores on the GBCA items, whereas the opposite was true for the NCD group. In addition, there were highly significant positive relationships between the GBCA (including the total score and its subtests), the MMSE, and the CASI (including its subtests). The GBCA correlated well with the CASI (r2 = 0.90, p < 0.001) and with the MMSE (r2 = 0.92, p < 0.001), indicating concurrent validity. The exceptions to these results were the lack of correlation between the GBCA Registration task certain items of the CASI (Short-term memory, Orientation, Language and Animal-name fluency; with p-values of 0.077, 0.145, 0.054, and 0.0078, respectively). - The psychometric characteristics of the tests in the GBCA battery were similar to the characteristics of the more conventional CASI and MMSE tests. The GBCA was consistent with the MMSE and CASI in detecting degenerative dementia, which suggests that the GBCA and its items produce a high quality assessment of cognitive function. - ROC curves: The GBCA cut-off of 75/76 corresponded to measurements of sensitivity, specificity, and area under curve of 85.1%, 91.5%, and 0.978, respectively. The positive predictive value was 91.9%, and the negative predictive value was 84.4%. Overall, the GBCA shows high internal consistency, except for the “Registration” item. |
| Kitchen and Cooking | Non-immersive Virtual Task (based on ADLs) | Gnosis  Executive Function Praxis | - Comparison with traditional screening tool (CDR, CASI, and MMSE) - Statistical analysis: Computed for each participant the mean time spent to complete a scenario, and the errors and mean time spent on each game activity in the first session. A Mann–Whitney U test was conducted on diagnosis level (MCI vs AD), residence and presence of diagnostic criteria for apathy (yes vs. no) as independent between-subject factors. | - Correlation analysis: AD participants took significantly longer to complete a game scenario compared to MCI participants (p = 0.004). Furthermore, the first time a scenario was played with the clinician (t0), AD participants were significantly slower in the gnosis (p = 0.002), executive functions (p = 0.046), and praxis activities (p = 0.006) compared to MCI participants, and made more errors in the praxis activity (p = 0.046) thus suggesting that the game assessment was sensitive to differences in the level of general cognitive impairment. |
| NL Puzzle Task | Serious Game Build (See: "Flow Free" commercial game) | Global  Visuomotor  Visuospatial  Executive Function  Visuo-constructional | - Comparison with traditional screening tool (MoCA). Patients with HD also completed MMSE, Trail Making Test A & B, SMT - Statistical analysis: Due to non-normally distributed data, differences between the participant groups (young (18-31), older (64-79), oldest (86-98), people with PD, people with HD) were performed using the non-parametric Kruskal–Wallis test followed by multiple comparison post hoc tests. To assess the *concurrent validity* of the NL puzzle task, associations between NL puzzle performance measures and neuropsychological test measures were tested by correlational analyses (Spearman rank correlation coefficients). | - Correlation analysis: compared to young and older adults, patients with HD were significantly slower in both total solving, mental planning, and MET (i.e., total time needed to manually connect paths in the maze). HD patients also made significantly more errors and excess moves when solving NL puzzles. Findings also show that MET and average velocity of these movements in the NL puzzle game were significantly different between young, older, and oldest adults. - Concurrent validity: game performance measures from the NL puzzle game were significantly correlated with performance on tests for visuomotor, visuospatial, executive, and visuoconstructional function. Game performance was also associated with global cognitive ability. Results indicate that game-based measures of cognitive and motor performance can be used to assess and monitor cognitive function in normal aging and neurodegenerative diseases. |
| Search and Match Task (SMT) | Gamified (combined a TMM3 puzzle video game with the visual search paradigm) | Visual Search | - Comparison with traditional screening tool (MoCA) - Comparison with test battery: pattern comparison task (PCT), visual scanning subtest, TMT: A & B - Statistical analysis: age-group differences in demographic variables, neuropsychological test measures, and SMT puzzle game performance measures were analysed using the nonparametric Kruskal-Wallis test, with subsequent pairwise Wilcoxon rank sum tests (using Bonferroni correction) for post hoc intergroup comparisons. Search slope was calculated by means of a general linear model (GLM). External validity was examined through correlation analyses (using the Spearman rank correlation coefficients) between the geometric mean search time and the performance on cognitive tests with measures of selective (TMT A completion time) and divided (TMT B completion time), visuospatial processing speed and pattern recognition (mean overall response time), and visual search (mean response time for target present trials). | - Correlation analysis: Time-based performance measures revealed significant age-group differences in overall completion time (χ2 2=337.6; P<.001), average target search time for all trials (χ2 2=374.1; P<.001), and trials without hints (χ2 2=330.3; P<.001) in the short puzzle game. Post hoc analyses for task completion time for the short difficulty level version showed that young adults (mean 5.34) were significantly faster compared with both older adults (mean 15.26; P<.001) and oldest adults (mean 21.99; P<.001). In addition, older adults were significantly faster than the oldest adults (P<.001). Regarding average search time, oldest adults (mean 8.39; mean 8.43) were significantly slower than both older adults (mean 4.32; mean 4.34; P<.001) and young adults (mean 2.75; mean 2.74, P<.001), and older adults were significantly slower than young adults (P<.001), respectively. - The GLM revealed a significant positive effect of set size (F1,2760=2.18; P=.01; Cohen f=.026), a significant negative effect of the number of tile types (F1,2760=8.17; P=.01; Cohen f=.05) and a significant positive effect of age (F1,2760=408.3; P<.001; Cohen f=.35) on target search time. For the long version of the game, the GLMEM analysis revealed a significant positive effect of set size (F1,7569=34.70; P<.001; Cohen f=.08), a significant negative effect of the number of tile types (F1,7569=35.86; P<.001; Cohen f=.081), and a significant positive effect of age (F1,7569=14.12; P<.001; Cohen f=.051) on target search time. - External validity: for the short puzzle difficulty level version, Spearman correlation analyses showed significant positive associations between geometric mean search time and TMT A completion time (r=.724; P<.001) and TMT B completion time (r=.755; P<.001). Furthermore, there was a significant negative correlation between geometric mean search time and the MoCA score (r=−.453; P=.01). To further evaluate the contribution of age on the neuropsychological tests, partial correlations of geometric mean search time with the neuropsychological test measures controlling for age were assessed. The partial correlation of both TMT A (r=.374; P=.02) and TMT B (r=.342; P=.03) completion time with geometric mean search time remained significant when controlling for age. However, the partial correlation between MoCA (controlling for age) and geometric mean search time was not significant (r=−.178; P=.27). |
| Smart Aging Serious Game (SASG) | Non-immersive Virtual Task (based on ADLs) | *Task 1:* Memory, spatial orientation  and attention  *Task 2:* Executive functions (planning), divided attention (dual task)  *Task 3:* Executive functions, selective attention, working memory, prospective memory  *Task 4:* Memory (recognition)  *Task 5:* Long-term memory (recall), spatial orientation and attention | - Comparison with traditional screening tool (MoCA) - Comparison with test battery: FCSRT, TMT: A & B - Statistical analysis: performed between groups (aMCI and HC) direct comparisons (one-way ANOVA or Mann–Whitney) in SASG gameplay to detect aMCI (based on MoCA total score, FCSRT scores). Also conducted ROC (Receiver Operating Characteristics) curve analysis to determine differences in the sensitivity and specificity of SASG in comparison with MoCA, FCSRT, TMT A and B. On the basis of our ROC curves, the best cut-off score for SASG in discriminating between HC and MCI was also investigated (Youden J index). | - Correlation analysis: Results show significant differences between groups in the accuracy of all SASG tasks, with the exception of T2 and T3, and in the time indices of all subtests except the T4. The SASG-total score (global performance aggregate) was significantly lower in the aMCI group. - ROC curves: The results show high values for both parameters for all tests except the TMT A and B. Moreover, the ROC comparison analysis reveals that SASG-total is comparable to MoCA and FCSRT in the ability to discriminate between groups, while the comparison with TMT A and B reveals significantly higher ability for the SASG-total. |
| Virtual Games | Non-immersive Virtual Task (similar multiple errand tasks, but performed in "VR") | IADLs; propose that activities of daily living should not be looked at by examining its constituent cognitive processes only, but also by how these work together, as the whole (i.e. IADL) may be more than the sum of its parts (i.e. cognitive processes). | - Previous clinical diagnosis of AD vs healthy control (healthy control cognitive health status confirmed by MoCA at study commencement) - Statistical analysis: independent-samples t-tests were used for group comparison for the time for each task and the time spent to ‘search the way’. To examine possible group differences in the binary response variable task achievement, separate logit-models per task were also computed. | - Correlation analysis: The independent-samples t-tests yielded a significant group difference in the mean time for the navigation tasks ‘Go to the Shop’ (t (36) = 12.457, p = .003), ‘Go back home’ (t (36) = .054, p = .005), whereas no significant differences were found for the navigation task ‘Go to the garden’ (t (36) = .001, p = .069) between the two groups. Furthermore, it revealed a significant group difference in the mean time for the shopping task (t (36) = 14.731, p < .001) and the cooking task (t (36) = 1.265, p < .05), indicating that patients needed more time to achieve these tasks compared to healthy controls, while no differences between the two groups were established for the mean time of the table preparation task (t (36) = 2.798, p = .321). The independent-samples t-tests on the time to search the way yielded a significant difference between the two groups (t (36) = 6.760 p = .01), signifying that patients needed significantly more time to search their way compared to the healthy controls. - The logit models did not reveal any relationship between education level and game performance (χ2(1) = 2. 61, p > 0.05) suggesting that the level of education did not influence performance. In addition, the logit-models revealed a significant difference between the two groups in the probabilities of achieving the cooking task (χ2(1) = 23.97, p < .001), the shopping task (χ2(1) = 20.00, p < .001), the navigation tasks ‘Go back home’ (χ2(1) = 13.49, p < .001) and ‘Go to the garden’ (χ2(1) = 31.76, p < 0.001) and the table preparation task (χ2 = 7.77, p < .01). |
| Virtual Supermarket Test (VST) | Non-immersive Virtual Task (based on ADLs | Executive Functions (main focus)  Visual and verbal memory  Attention  Spatial Navigation | - This study focuses on assessing the VST’s usability in a sample of older adults with MCI and SCD. Psychometric validity testing of the VST is not undertaken in the included study, however, validity testing has been conducted in the VST numerous times and it has been shown to outperform the MoCA in differentiating between MCI patients and healthy controls [15, 47-48]. For instance, in a recent study of the VST [15], a correct classification rate (CCR) of 81.91% when differentiating between MCI patients and older adults with SCD, while the MoCA displayed of CCR of 72.04% and the MMSE displayed a CCR of 64.89%. |  |

**References**

15. Zygouris S, Iliadou P, Lazarou E, Giakoumis D, Votis K, Alexiadis A, et al. Detection of mild cognitive impairment in an at-risk group of older adults: can a novel self-administered serious game-based screening test improve diagnostic accuracy? J Alzheimers Dis. 2020;78(1):405-412 [doi: 10.3233/JAD-200880] [Medline: 32986676]

47. Paliokas I, Kalamaras E, Votis K, Doumpoulakis S, Lakka E, Kotsani M, et al. Using a virtual reality serious game to assess the performance of older adults with frailty. Adv Exp Med Biol. 2020;1196:127-139. [doi: 10.1007/978-3-030-32637-1_13] [Medline: 32468314]

48. Zygouris S, Giakoumis D, Votis K, Doumpoulakis S, Ntovas K, Segkouli S, et al. Can a virtual reality cognitive training application fulfill a dual role? Using the virtual supermarket cognitive training application as a screening tool for mild cognitive impairment. J Alzheimers Dis. 2015;44(4):1333-1347. [doi: 10.3233/JAD-141260] [Medline: 25428251]
