# Supplementary material for: Evaluating the User Experience and Usability of Game-Based Cognitive Assessments for Older People: Systematic Review
Source: JMIR Aging. 2025 Jun 11;8:e65252. doi: 10.2196/65252 (PMC12198696; doi:10.2196/65252)
Supplement: Multimedia Appendix 4 [file aging_v8i1e65252_app4.docx]

| **Appendix 4: summary of user evaluation findings** | | |
| --- | --- | --- |
| Game | Method(s) | Evaluation findings |
| Episodix | - User-experience questionnaire based on the Technology Acceptance Model. - Focus Groups to understand participant preferences. | - User-experience questionnaire: 5-point Likert scale questionnaire, ranging from strongly disagree (1 point) to strongly agree (5 points). The questionnaire is based on the TAM (Technology Acceptance Model) [51], which is considered adaptable to senior adult cohorts [52]. The TAM indicates that the attitude towards a given technological system is determined by two key variables; perceived ease of use and perceived usefulness. The questionnaire helped to capture participants reports of these two variables before and after playing the game.   - The motivation and the intention to interact with games and the perceived usefulness of games dramatically increased after interacting with Episodix. Before using the digital game, participants reported having a very low motivation, with the HC group obtaining the lowest score. This value increased to almost 40% after the pilot experiment.   - All the participants perceived that the use of video games for cognitive assessment seemed helpful. In addition, this value increased after the pilot experiment, especially in the control group (i.e., a 30% increase).   - Participants rated their intention to use the digital game at an average rating of 3 at the beginning, and of 4 at the end of pilot experiment.   - Participants claimed that they would play much more with video games (40% increase for HC, 20% for MCI and 40% for AD).   - Participants indicated that the Episodix game seemed easier than traditional tests, in particular the CVLT, and they suggested it was more engaging and less intrusive than the pen-and-paper test (ie, a 4 out of 5 median values).   - The significance of the increase between pre- and post-pilot perceptions was measured by a paired samples t-test. The main outcomes showed that this increase is significant in willingness to play more video games (t D0:000426); motivation to use video games (t D0:001291); and finally, perception of considering this kind of cognitive game as a useful tool (t D0:040568). - Focus Group participants had a negative initial perception about information technologies in general, and video games in particular. This perception dramatically improved when they were introduced to the game and they realized its cognitive utility. |
| Game-Based Cognitive Assessment (GBCA) | - User-experience questionnaire, focusing on ease-of-use, game narrative, user interface and comparison with traditional tests. | - The user-experience questionnaire included one question on game ease-of-use, two questions about whether users felt the game stories were interesting and familiar, seven questions about users’ reactions to the interface design (typesetting, color, instruction, etc.) and four questions aimed at comparing the GBCA with other cognitive assessment tools (clear enough to understand, easy to use, etc.). Responses to each question were recorded on a 5-point scale (5 = very satisfied, 4 = satisfied, 3 = neutral, 2 = unsatisfied, and 1 = very unsatisfied).   - The responses of the HC group on all items of the questionnaire ranged from 4 to 5, suggesting they were quite satisfied with their experience of using the app, and they thought it was easy to operate, well designed, and useful as a self-assessment tool.   - The average response of the NCD group was between 3.3 and 3.7, which puts their level of satisfaction with the app somewhere between “neutral” and “satisfied”.   - Comparisons from the questionnaire showed a significant difference between the HC and NCD groups. The HC group was more satisfied with the GBCA than the NCD group.   - Lower scores given by participants to the user-experience questionnaire corresponded to a higher CDR score, a higher age, and a lower education level. More specifically, the severity of cognitive impairment adversely affected the participants’ ability to use the tablet to complete the GBCA, as suggested by the poor ratings given to the text and design of the GBCA.   - Age specifically had a negative influence on the participants’ satisfaction with the pictures, text, and story used in the GBCA. Similarly, education level influenced, somewhat negatively, the participants’ satisfaction with the pictures and text. |
| Kitchen and Cooking | - User-experience questionnaire,   focusing on satisfaction, interest, motivation, emotional experience and fatigue. | - At two points during the study participants completed a self-report user-experience questionnaire concerning game experience. Satisfaction was assessed through a 10 cm analogical scale, in which participants were asked to bisect a line ranging from ‘not satisfied at all’ to ‘really satisfied’. Interest was assessed through a 4-item 1–7 Likert scale adapted from Gourlan et al. [53]. Motivation was evaluated through an adaptation of the scale proposed by Gourlan et al. [53], a 24-item 0–7 Likert scale which measures separately intrinsic motivation (e.g., “I play because it is fun”) and external motivation (“I play because my friends/family members say I should”). Emotional experience of gameplay was assessed through the PANAS scale [54], a 20-item 0–5 Likert scale evaluating separately self-reported positive and negative emotions. Fatigue was evaluated through the French adaptation of the Piper Fatigue Scale (11 rating questions, scale 0–10; [55]).   - The results of the self-report questionnaires (mean scores between both sessions) showed that, as a group, participants reported to be highly satisfied with the overall game experience (Mean = 8.2/10, SD = 1.3), they were interested in the game (Mean = 17.1/28, SD = 5.6), and motivated by the activity.   - Intrinsic motivation (Mean = 3.9/7, SD = 1.3), was significantly higher than external motivation [Mean = 2.5/7, SD = 1.2; t(15) = 4.37, p = 0.001].   - Participants reported to not be very fatigued (Mean = 3.7/10; SD = 1.2).   - Users experienced more positive emotions (PANAS pos, M = 2.7/5; SD = 0.8) than negative emotions [PANAS neg, M = 1.4/5, SD = 0.6, t(18) = 5.86, p < 0.001].   - The interest, satisfaction, motivation (intrinsic and extrinsic), fatigue and emotions scores (positive and negative) did not change between the two sessions (t ranging from 0.15 to 1.8, p from 0.092 to 0.880).   - AD participants reported to be significantly more satisfied compared to the MCI participants (p = 0.043).   - Apathetic participants reported to experience fewer positive emotions (p = 0.008) compared to nonapathetic participants.   - No difference in the self-report scales was found between outpatients and patients living in nursing homes (all p > 0.323). |
| NL Puzzle Task | - User-experience was assessed using the Perception of Game Training Questionnaire [50]. - System Usability Scale (SUS) was used to measure user experience, usability, and learnability. | - Subjective user experience and acceptance of the NL puzzle game was assessed with the Perception of Game Training Questionnaire [50]. In this questionnaire, participants rated the extent to which they found playing the mazes enjoyable, challenging, frustrating, as well as their motivation levels while playing on a seven-point Likert scale.   - Average *enjoyability rating* across the groups were as followed: YA (6.28 ± 0.75), OA (6.14 ± 1.56), OOA (6.64 ± 0.50), PD (6.50 ± 0.58), HD (5.80 ± 1.10). Average *challenge rating* across the groups were as followed: YA (2.78 ± 1.56), OA (3.86 ± 1.61), OOA (2.86 ± 1.92), PD (5.00 ± 1.41), HD (4.20 ± 2.17). Average *frustration rating across* the groups were as followed: YA (1.00 ± 0.00), OA (1.29 ± 0.83), OOA (1.29 ± 0.47), PD (1.50 ± 0.58), HD (1.60 ± 0.89). Average *motivation rating* across the groups were as followed: YA (6.56 ± 0.62), OA (6.64 ± 0.50), OOA (6.64 ± 0.63), PD (7.00 ± 0.00), HD (6.60 ± 0.55).   - Ratings of enjoyment (χ 2 (4) = 3.81; p = 0.43), challenge (χ 2 (4) = 8.75; p = 0.07), frustration (χ 2 (4) = 8.98; p = 0.06), and motivation (χ 2 (4) = 2.36; p = 0.67) for the NL puzzle game did not differ significantly between groups. - The 10-item System Usability Scale (SUS) was used to measure user experience, usability, and learnability of the NL puzzle game. The SUS provides a composite score from 0 to 100 where a higher number indicates a higher usability.   - The SUS averages across the groups were as followed: YA (93.38 ± 5.72), OA (93.33 ± 6.25), OOA (83.39 ± 11.79), PD (81.88 ± 9.66), HD (83.12 ± 10.68). Overall, individual SUS ratings ranged from 67.50 (‘‘good’’) to 100.00 (‘‘best’’) with a mean SUS rating of 86.43 (‘‘excellent’’).   - There was a significant difference in usability ratings between groups (χ 2 (4) = 12.04; p = 0.02), but post hoc comparisons failed to show any significant differences between groups (YA vs. OA, OOA vs. PD, OOA vs. HD, PD vs. HD: p = 1.000, YA vs. OOA p = 0.140, YA vs. PD p = 0.609, YA vs. HD p = 0.355, OA vs. OOA p = 0.228, OA vs. PD p = 0.689, OA vs. HD p = 0.414). |
| Search and Match Task (SMT) | - User-experience was assessed using the Perception of Game Training Questionnaire [50]. - System Usability Scale (SUS) was used to measure user experience, usability, and learnability. | - Similar to the NL Puzzle Task, user experience and acceptance was assessed with the Perception of Game Training Questionnaire [50]. Participants rated the extent to which they found playing the SMT enjoyable, challenging, and frustrating as well as their motivation while playing the mazes on a seven-point Likert scale.   - Average (SD) *enjoyability rating* across the groups were as followed: Young adults = 5.32 (1.09), Older = 5.92 (0.86), Oldest = 6.10 (1.60); p = 0.12. Average (SD) *challenge rating* across the groups were as followed: Young adults = 4.00 (1.68), Older = 5.62 (1.04), Oldest = 5.50 (0.97); p = 0.01. Average (SD) *frustration rating* across the groups were as followed: Young adults = 2.11 (1.45), Older = 2.54 (1.61), Oldest = 2.30 (2.21); p = 0.74. Average (SD) *motivation rating* across the groups were as followed: Young adults = 5.96 (1.07), Older = 6.31 (0.48), Oldest = 6.50 (0.97); p = 0.25. Average (SD) *difficulty rating* (*SHORT* puzzle version) across the groups were as followed: Young adults = 2.69 (1.62), Older = 3.34 (1.48), Oldest = 3.43 (2.24); p < 0.001. Average (SD) *difficulty rating* (*LONG* puzzle version) across the groups were as followed: Young adults = 2.82 (1.68), Older = 3.43 (1.69), Oldest = N/A; p < 0.001.   - In terms of perception of the SMT, there were significant differences between the three age groups regarding ratings of challengingness (χ2 2=10.2; P<.001). On the whole, young adults (mean 4.00) perceived the SMT as significantly less challenging than older adults (mean 5.62; P=.02).   - There were no significant group differences in terms of enjoyment, frustration, and motivation while playing the SMT task.   - Regarding average difficulty rating based on ratings for each difficulty level, there were significant age group differences in average difficulty ratings for both the short version played by the young, older, and oldest adults (χ2 2=266.6; P<.001) and the long version (χ2 2=479.4; P<.001) played by the young and older adults.   - Average difficulty ratings for all played levels in the short version revealed that younger adults rated these difficulty levels as significantly less difficult (mean 2.69) than both the older (mean 3.34; P<.001) and oldest adults (mean 3.43; P<.001).   - Average difficulty ratings for all levels in the long version further showed that the young adults (mean 2.82) gave significantly lower difficulty ratings than the older adults (mean 3.43; P<.001). - The 10-item SUS was used to measure user experience, usability, and learnability of the NL puzzle game.   - Average (SD) SUS scores across the groups were as followed: Young adults = 88.67 (7.28), Older = 79.09 (15.50), Oldest = 68.25 (18.78); p < 0.01   - Overall system usability ratings for the SMT indicated a significant effect of age group (χ2 2=10.4 P<.01). Oldest adults (mean 68.25) ranked the usability significantly lower than young adults (mean 88.61; P=.007). Individual usability ratings ranged from 72.50 (good) to 97.50 (excellent) in young, from 52.50 (okay) to 100 (excellent) in older, and from 32.50 (unacceptable) to 95.00 (excellent) in oldest adults. |
| Smart Aging Serious Game (SASG) | - Computer familiarity questionnaire focusing on computer/touch screen total use and frequency of use. | - Participant computer familiarity was collected based on a previously designed computer usage scale [56]. Specifically, participants were asked questions concerning their familiarity with computers and touch-screen use, expressed in terms of frequency of use, before the SASG session.   - Results from the computer familiarity scale revealed no differences between groups in regard to the frequency of computer use, with 53.12% of aMCI and 60.38% of HC subjects reporting rarely using a PC, while the remaining participants had a frequent use (at least weekly).   - The frequency of use of a touch screen was also comparable between groups: 62.5% of aMCI and 72.65% of HC had never used a touch screen before the participation in the study; 18.75% of aMCI and 20.75% of HC who used it unfrequently (not more than once a month); and 18.75% of aMCI and 6.60% of HC who had a frequent (at least weekly) use.   - In order to test the influence of the familiarity with computers and the SASG score, a 2 x 2 ANOVA with clinical group (aMCI vs HC) and frequency of computer use factor (infrequent vs. frequent) on SASG total score data was performed. Testing the influence of familiarity on SASG score, ANOVA results showed a significant group effect (F(1134) = 64.109, p < 0.001), however, the effect of frequency of use factor was not significant (F(1134) = 2.975, p = 0.087) and no significant interactions were found (F(1134) = 0.74, p = 0.391), indicating that familiarity with the use of the PC did not influence SASG score. |
| Virtual Games | - System Usability Scale (SUS) was used to measure user experience, usability, and learnability. - User gameplay data, to examine differences in velocity, curvature, length of movements, and number of movements | - The SUS was used as a subjective measure of usability.   - The mean SUS score for the control group was 83.5 ± 11.16. The mean SUS score for the AD patient group was 83.75 ± 9.82. Revealing no significant subjective usability differences reported across groups. - Data collected from user computer interactions during gameplay was used as a measure of, what the authors referred to as, objective usability.   - An independent-samples t-test was used to assess potential differences in the objective measures of usability (i.e. differences in velocity, curvature, length of movements, and number of movements) and the mean score of usability with the SUS. The nature of interactions was equivalent in healthy controls and AD patients, and the authors concluded that the difference in performance was not influenced by the usability. An ANOVA did not reveal any significant effect of score (p = .806), indicating that both groups found the game user friendly. |
| Virtual Supermarket Test (VST) | - System Usability Scale (SUS) was used to measure user experience, usability, and learnability. - Computer familiarity question assessing touch device usage. | - The SUS was used in addition to a further binary question regarding familiarity with touch devices where participants were instructed to answer “Yes” if they could operate the touch device and do simple tasks (e.g., find a specific contact in the contacts app) unassisted.   - The average SUS score was 83.11 (SD = 14.6). There were no significant differences found between the SCI and MCI groups in SUS scores. Similarly, no significant differences in SUS scores were found between participants with touch device familiarity and participants without touch device familiarity.   - Significant correlation (r = –0.496, p = 0.000) was found between SUS score and average time needed to complete the 3 VST test trials.   - No significant correlations were found between SUS score, age, education and total mistakes conducted during the 3 VST test trials. |

**References**

50. Boot WR, Champion M, Blakely DP, Wright T, Souders DJ, Charness N. Video games as a means to reduce age-related cognitive decline: attitudes, compliance, and effectiveness. Front Psychol. 2013;4:31. [doi: 10.3389/fpsyg.2013.00031] [Medline: 23378841]

51. Lee Y, Kozar KA, Larsen KR. The technology acceptance model: past, present, and future. Commun Assoc Inf Syst. 2003;12:50. [doi: 10.17705/1cais.01250]

52. Rivas Costa C, Fernández Iglesias MJ, Anido Rifón LE, Gómez Carballa M, Valladares Rodríguez S. The acceptability of TV-based game platforms as an instrument to support the cognitive evaluation of senior adults at home. PeerJ. 2017;5:e2845. [doi: 10.7717/peerj.2845] [Medline: 28070464]

53. Gourlan M, Sarrazin P, Trouilloud D. Motivational interviewing as a way to promote physical activity in obese adolescents: a randomised-controlled trial using self-determination theory as an explanatory framework. Psychol Health. Nov 2013;28(11):1265-1286. [doi: 10.1080/08870446.2013.800518] [Medline: 23756082]

54. Watson D, Clark LA, Tellegen A. Development and validation of brief measures of positive and negative affect: the PANAS scales. J Pers Soc Psychol. Jun 1988;54(6):1063-1070. [doi: 10.1037//0022-3514.54.6.1063] [Medline: 3397865]

55. Gledhill JA, Rodary C, Mahé C, Laizet C. [French validation of the revised Piper Fatigue Scale]. Rech Soins Infirm. Mar 2002;(68):50-65. [Medline: 12001628]

56. Bottiroli S, Cavallini E. Can computer familiarity regulate the benefits of computer-based memory training in normal aging? A study with an Italian sample of older adults. Neuropsychol Dev Cogn B Aging Neuropsychol Cogn. Jul 29, 2009;16(4):401-418. [doi: 10.1080/13825580802691763] [Medline: 19253069]
